# Dual Effects of Metformin on Adipogenic Differentiation of 3T3-L1 Preadipocyte in AMPK-Dependent and Independent Manners

**DOI:** 10.3390/ijms19061547

**Published:** 2018-05-23

**Authors:** Dian Chen, Ying Wang, Kaikai Wu, Xingya Wang

**Affiliations:** College of Pharmaceutical Science, Zhejiang Chinese Medical University, 548 Binwen Road, Hangzhou 310053, China; chendian_zcmu@163.com (D.C.); wangying19870523@163.com (Y.W.); 15990099051@163.com (K.W.)

**Keywords:** metformin, adipogenesis, MAPKs, AMPK, 3T3-L1 preadipocyte

## Abstract

Metformin has been reported to have body weight lowering effects while treating type 2 diabetes. However, limited studies examined the effects of metformin on adipogenesis in vitro, and available data are inconclusive and contradictory. In this study, we examined the effects of a variety of concentrations of metformin on adipocyte differentiation of 3T3-L1 preadipocytes and found metformin exhibits a dual effect on adipogenesis. Metformin at lower concentrations (1.25–2.5 mM) significantly induced adipogenesis while at higher concentrations (5–10 mM) metformin significantly inhibited adipogenesis in 3T3-L1 cells. The biphasic effect of different doses of metformin on adipogenesis was accompanied by increasing or decreasing the expression of adipogenic and lipogenic genes including peroxisome proliferator-activated receptor (PPARγ), CCAAT/enhancer binding protein α (C/EBPα), and fatty acid synthase (FASN) at both messenger RNA (mRNA) and protein levels. Furthermore, only the higher concentrations of metformin induced the phosphorylation of adenosine 5′-monophosphate (AMP)-activated protein kinase (AMPK), p38, and c-Jun N-terminal kinase (JNK) and reduced the phosphorylation of extracellular regulated protein kinases (ERK) and Akt. Pretreatment with compound C, a specific AMPK inhibitor, significantly countered high concentration of metformin-induced inhibition of adipogenesis. Taken together, these findings demonstrate that the effect of metformin on adipocyte differentiation is biphasic and dose-dependent. Lower concentrations of metformin induce adipogenesis, which could be mediated in an AMPK-independent manner, while higher concentrations of metformin inhibit adipogenesis via AMPK activation.

## 1. Introduction

Obesity is a worldwide epidemic as the prevalence of obesity among the adult population has increased rapidly in both developed and developing countries. The rise in obesity has also led to a concurrent rise in type 2 diabetes mellitus (T2DM), cardiovascular disease, and cancer [1]. Obesity is characterized by an excessive accumulation of fat in adipose tissue as well as in the liver, muscles, and other organs. Adipose tissue is a complex organ that regulates and coordinates energy homeostasis [2]. It has been well-established that excessive adipogenesis leads to obesity. According to the World Health Organization, an estimated 422 million adults worldwide (8.5%) are affected by T2DM, and this number is expected to rise to 592 million by 2035 [3]. It is worth noting that a majority of patients with T2DM are obese, and obese people are at a higher risk of developing T2DM [4].

Biguanide metformin has long been used for its glucose-lowering effects and insulin sensitivity-improving activities. Metformin has also gained attention for its pleiotropic effects, which include decreasing appetite and food intake, preventing cardiovascular disease, improving endothelial function, modulating inflammation, preventing cancer, and in particular, lowering the body weights of T2DM patients [5]. Due to its body weight lowering effects, metformin has been the first-line therapeutic option for the treatment of obese T2DM patients [6]. Ironically, although weight loss is often viewed as a favorable “side-effect” of metformin, very little data show weight loss in nondiabetic metformin patients [7]. A significant number of clinical trials suggest that metformin is less effective in persons with lower baseline body mass index (BMI) than in those with higher BMIs who are considered to be obese [8]. However, the underlining mechanism for why metformin is more effective in lowering body weights in obese T2DM patients is unknown. Furthermore, conflicting data concerning the anti-diabetic and weight loss actions of metformin indicate that metformin’s effects are dose-dependent, particularly at high dosages (>1500 mg/day) [9]. 

Although clinical results suggest that metformin has a weight-lowering effect, there are very limited data available from in vitro or in vivo studies examining the effects of metformin on adipogenesis and its molecular targets. Effects of metformin have been evaluated in the murine 3T3-L1 cell line, in which several investigators found an inhibition of adipogenesis by metformin [10,11,12]. However, most of these studies only examined a single dose of metformin and found metformin inhibited adipogenesis at or above a 4∼5 mM dose, but very few studies examined concentrations at the nmol or µmol range. One study reported that metformin at >4 mM significantly reduced lipid accumulation in 3T3-L1 cells, while 2 mM of metformin increased adipogenesis without statistical significance [10]. It was estimated that the plasma concentration of metformin may vary from 1 to 50 µM in mice given 50 mg/kg/day of metformin [13,14], which is far less than concentrations used in in vitro studies. Frid et al. reported that the maximal serum level of metformin in T2DM patients after consuming 500–3000 mg/day is approximately 20 µM [15]. Discrepancies between the concentrations of metformin used in in vitro studies versus at therapeutic levels indicate that it is necessary to examine the effects of metformin on adipogenesis at lower concentrations in in vitro systems. Furthermore, adipogenesis is a complex process that involves a cascade regulation of transcription factors and genes associated with lipogenesis as well as a network coordination of signaling transduction pathways [2]. However, there is very limited research on the effects of metformin on regulating transcriptional factors or signaling pathways during adipogenesis.

In this study, we examined the dose effects and molecular mechanisms of metformin on adipogenesis in mouse 3T3-L1 preadipocytes, which is a well-established model system for studying adipogenic differentiation. We found that at the nmol and µmol range, metformin has no effects on 3T3-L1 differentiation. However, at concentrations above 1 mM, metformin exhibits a biphasic effect on adipogenesis that at lower concentrations (1.25 and 2.5 mM) induces, but at higher concentrations (5 and 10 mM) reduces adipocyte differentiation. Further studies revealed that the induction of adipogenesis by lower concentrations of metformin might not be mediated by AMPK signaling, while the inhibition of adipogenesis by higher concentrations of metformin may be dependent on AMPK activation. This is the first study that thoroughly examined the effects of a wide range of doses of metformin on adipogenesis in vitro. Our results suggest a complex role of metformin in adipogenesis, and the dose of action of metformin should be considered in future studies. 

## 2. Results

### 2.1. Effects of Various Concentrations of Metformin on Differentiation and Lipid Accumulation in 3T3-L1 Preadipocytes

**Figure 1:**
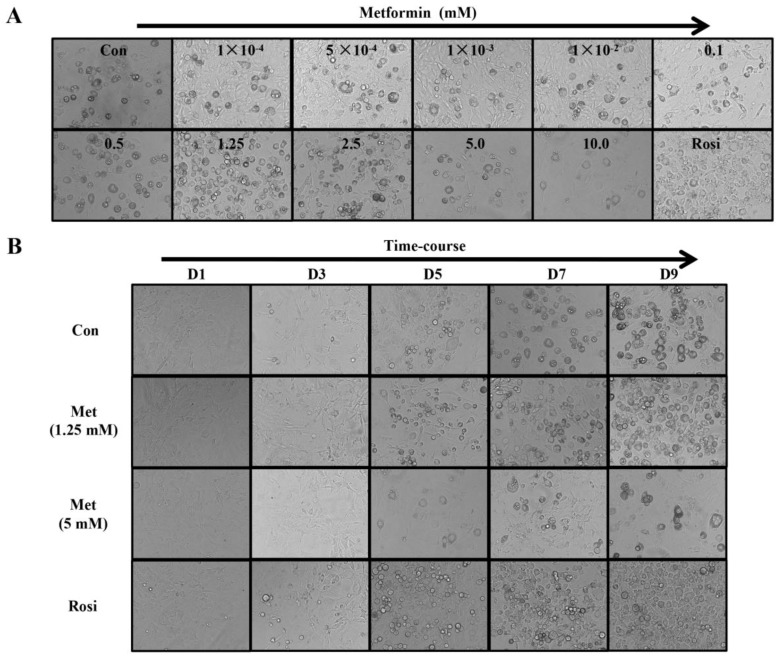
Effects of metformin at various concentrations on 3T3-L1 preadipocytes differentiation. (**A**) Effects of metformin (Met) on adipogenesis in a dose-dependent manner. The 3T3-L1 preadipocytes were treated with a differentiation cocktail and various doses of metformin for 9 days as observed by a microscope, 200×; (**B**) Time course of low or high concentrations of metformin (1.25 or 5 mM) on adipogenesis at day 1 (D1), 3 (D3), 5 (D5), 7 (D7), and 9 (D9) as observed by a microscope, 200×. Control cells were treated with differentiation medium I (DMI) only. Rosiglitazone (Rosi, 2.5 µM) was used as positive control.

We first examined whether metformin has an influence on cell proliferation of 3T3-L1 preadipocytes. A (3-(4,5-dimethylthia-zol-2-yl)-2,5-diphenyltetrazolium bromide) (MTT) assay revealed that metformin had no cytotoxicity to 3T3-L1 cells at concentrations lower than 20 mM, however, 30 mM of metformin significantly inhibited cell viability. Next, we determined the effects of various concentrations of metformin on adipocyte adipogenesis using concentration lower than 20 mM. As shown in Figure 1A, at the end of differentiation, lower concentrations of metformin at 100 and 500 nM, and 1, 10, 100, and 500 μM showed no significant effects on the differentiation of 3T3-L1 cells. Interestingly, at concentrations above 1 mM, metformin demonstrated biphasic effects on adipogenic differentiation. At 1.25 and 2.5 mM, metformin significantly increased adipocyte differentiation, while 5 and 10 mM of metformin significantly reduced adipocyte differentiation (Figure 1A). During the 9-day differentiation period, obvious adipocyte formation was observed at day 5 (D5), during which the biphasic effects of metformin continued from D5 to D9 (Figure 1B). Rosiglitazone (2.5 μM) was used as a positive control, which dramatically induced adipogenic differentiation and was more potent than 1.25 or 2.5 mM of metformin in inducing adipogenesis in 3T3-L1 cells. 

**Figure 2:**
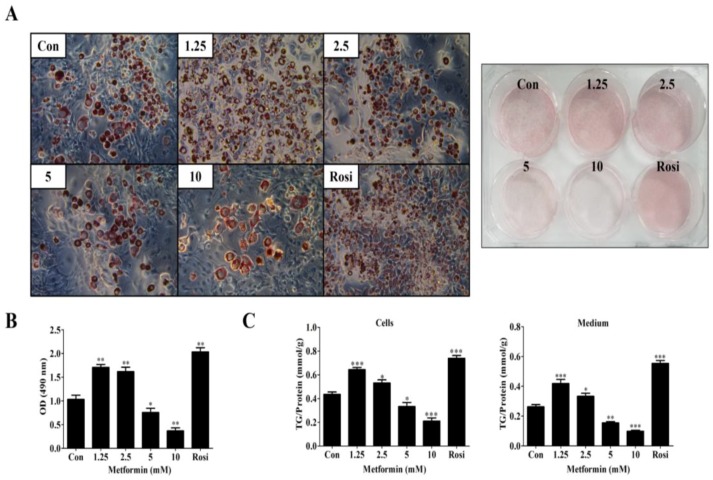
Effects of different concentrations of metformin (1.25–10 mM) on lipid accumulation in 3T3-L1 cells. (**A**) Effects of metformin on adipocyte formation at day 9 as determined by Oil Red O staining, 100×. Microscopic observation (**left**); 6-well plate (**right**); (**B**) Optical density (OD) of quantified Oil Red O staining in 3T3-L1 cells (*n* = 3); (**C**) Effects of metformin on intracellular and extracellular TG contents at day 9 as determined by triglyceride GPO-POD (glycerophosphate oxidase-phenol aminophenazone) enzymatic assay. Results are normalized by protein concentration in cell lysates (*n* = 3). The data represents the means ± SEM (standard error of mean) from three independent experiments. * *p* < 0.05, ** *p* < 0.01 and *** *p* < 0.001 as compared with DMI alone treated cells (one-way analysis of variance (ANOVA) with Dunnett’s correction).

To further evaluate the effects of metformin on lipid accumulation in 3T3-L1 cells, adipocytes were visualized by Oil Red O staining and the dye retained in the cells was dissolved by iso-propyl alcohol (IPA) after a 9-day differentiation. As shown in Figure 2A, 1.25 and 2.5 mM of metformin increased the intensity of Oil Red O staining, but 5 and 10 mM of metformin reduced the intensity of staining compared to control cells (DMI alone). The biphasic effects of metformin were confirmed after quantification of Oil Red O staining (Figure 2B). In addition, 1.25 and 2.5 mM of metformin increased both the intracellular and extracellular concentrations of triglyceride (TG) contents compared to control cells (DMI alone) as normalized by total protein concentrations (Figure 2C). On the contrary, 5 and 10 mM of metformin significantly reduced both the intracellular and extracellular TG contents compared to control cells (Figure 2C).

### 2.2. Effects of Metformin on Expression of Adipogenic and Lipogenic Genes in 3T3-L1 Cells

**Figure 3:**
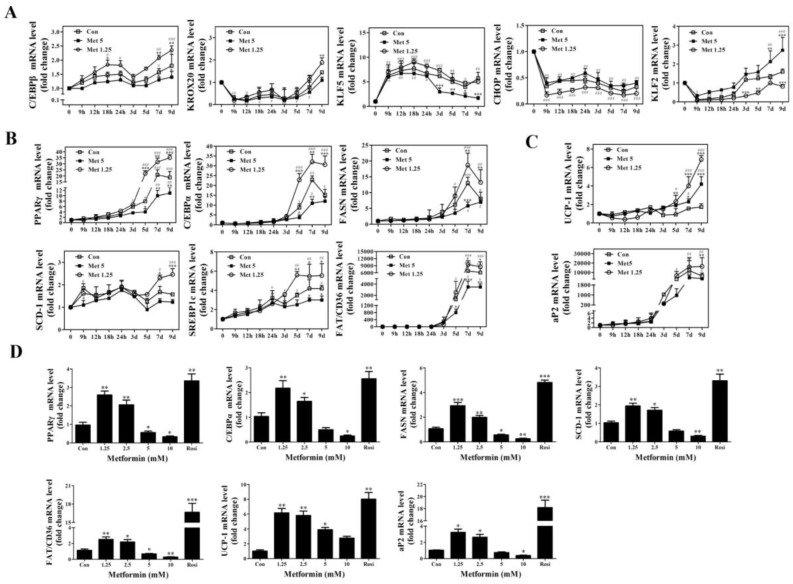
Effects of 1.25 or 5 mM of metformin on expression of adipogenic and lipogenic related genes. (**A**) Time course of gene expression upon treatment for marker genes during early stage of adipocyte differentiation (*n* = 6); (**B**) Time course of gene expression upon treatment for marker genes during late stage of adipocyte differentiation (*n* = 6). Post-confluent 3T3-L1 cells were treated with DMI mixture alone (Con) or DMI with metformin (Met: 1.25 or 5mM) for different times (0, 9, 12, 18, 24 h, 3, 5, 7, 9 day) and gene expression was determined by qRT-PCR; (**C**) Effects of metformin on expression of UCP-1 and aP2 during the differentiation of 3T3-L1 cells (*n* = 6, duplicate for each time and repeated 3 times); Data represents the means ± SEM from three independent experiments. Two-way ANOVA with post hoc Bonferroni’s correction for multiple comparison was used to determine statistical significance. “*” indicates treatment effects, and “^#^” indicates time effects. * *p* < 0.05, ** *p* < 0.01, and *** *p* < 0.001 as treatments compared with DMI alone treated cells at each time point. ^#^ *p* < 0.05, ^##^ *p* < 0.01, and ^###^ *p* < 0.001 as significant across time compared to control for each treatment; (**D**) Dose-dependent effects of metformin on adipogenic and lipogenic genes at day 9 after initiation of differentiation (*n* = 6). Data represents the means ± SEM from three independent experiments. * *p* < 0.05, ** *p* < 0.01, and *** *p* < 0.001 as compared with DMI alone treated cells (one-way ANOVA with Dunnett’s correction).

Adipogenesis is a complex process that involves a cascade of transcription factors regulating differentiation and a network of gene expression associated with lipogenesis [2]. In addition, the differentiation of preadipocytes into mature adipocytes usually takes place in several stages, from early to late, which can be regulated by different transcriptional factors [16]. We examined the effects of metformin on the expression of transcription factors at different time points (0, 9, 12, 18, 24 h; 3, 5, 7, 9 day) during adipocyte differentiation upon treating with low (1.25 mM) or high (5 mM) concentrations of metformin. Our data shows a trend of 1.25 mM of metformin inducing pro-adipogenic genes (CCAAT/enhancer binding protein β (*C/EBPβ*), *KROX20*, Krüppel-like Factor 5 (*KLF5*), peroxisome proliferator-activated receptor (*PPARγ*), CCAAT/enhancer binding protein α (*C*/*EBPα*), fatty acid translocase (*FAT*)/*CD36*, and sterol regulatory element-binding protein 1c (*SREBP1c*)) but reducing anti-adipogenic genes (transcription factor homologous to CCAAT-enhancer binding protein (*CHOP*) and Krüppel-like Factor 2 (*KLF2*)) (Figure 3A,B). In addition, 1.25 mM of metformin also induced the expression of lipogenic gene stearoyl-CoA desaturase-1 (*SCD-1*) and fatty acid synthase (*FASN*) (Figure 3B). Conversely, 5 mM of metformin demonstrated opposite effects such as inhibiting pro-adipogenic and lipogenic genes, but increasing anti-adipogenic genes (Figure 3A,B). Indeed, we found that early stage genes were induced or reduced at earlier time points (before 24 h) upon metformin treatment or DMI alone treatment, but late stage genes were induced or inhibited at later time points (5 day or after). To investigate the potential of metformin to lead to adipocytes “browning”, we also examined the expression of thermogenesis markers uncoupling protein 1 (*UCP-1*) and adipocyte Protein 2 (*aP2*), and found that *UCP-1* messenger RNA (mRNA) was significantly elevated by 1.25 and 5 mM of metformin during late stage of differentiation of 3T3-L1 cells compared with controls, whereas the expression of *aP2* was slightly reduced by metformin at 5 mM (Figure 3C). These data suggest that metformin may play a role in thermogenesis, however, further studies are needed to establish this association. 

We further examined the expression pattern of the late stage adipogenic and lipogenic genes in 3T3-L1 cells upon stimulation with metformin at various concentrations at D9. As shown in Figure 3D, lower concentrations of metformin (1.25 and 2.5 mM) significantly induced *PPARγ*, *C*/*EBPα*, *FASN*, *FAT*/*CD36*, *SCD-1*, and *aP2* expression, while higher concentrations of metformin (5 and 10 mM) inhibited the expression of these genes. These data correlate with the biphasic effects of metformin on adipogenic differentiation. However, all concentrations of metformin induced *UCP-1* expression (Figure 3D). Collectively, our results suggest that metformin has a dual effect on adipogenic and lipogenic gene expression dependent upon metformin dosage.

### 2.3. Effects of Metformin on FASN, C/EBPα, and PPARγ Protein Expression in 3T3-L1 Cells

**Figure 4:**
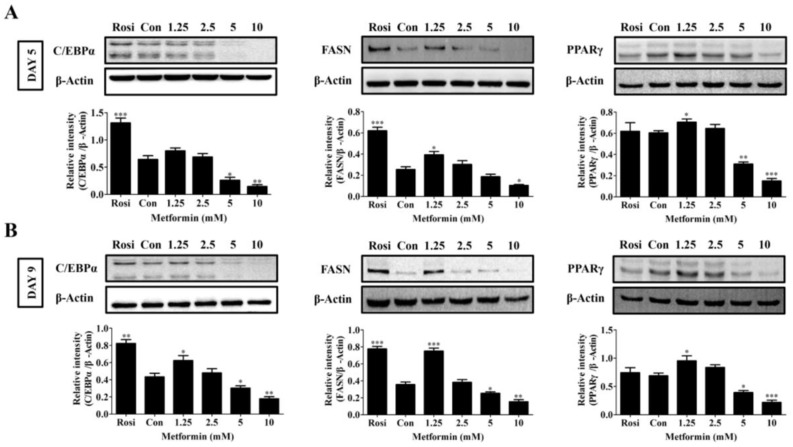
Effects of different doses of metformin (1.25–10 mM) on the expression of adipogenesis markers FASN, PPARγ, and C/EBPα in 3T3-L1 cells. (**A**) Expression levels at day 5 (DAY 5) after differentiation initiation as determined by Western blot analysis (*n* = 3); (**B**) Expression levels at day 9 (DAY 9) (*n* = 3). Rosiglitazone (Rosi, 2.5 µM) was used as a positive control. Results were quantified using densitometry analysis and normalized to β-Actin. The data represent the means ± SEM from three independent experiments. * *p* < 0.05, ** *p* < 0.01, and *** *p* < 0.001 as compared with DMI alone treated cells (one-way ANOVA with Dunnett’s correction).

During adipogenic differentiation and lipogenesis, late stage genes C/EBPα and PPARγ and lipogenic gene FASN are the most important adipogenic markers. We next evaluated the effects of metformin on the expression of these three genes at protein levels at day 5 and day 9 of differentiation. Consistent with our results on gene expression, the results showed that metformin also demonstrates biphasic effects on the expression of FASN, C/EBPα, and PPARγ at the protein level, with lower concentrations (1.25 and 2.5 mM) increasing and higher concentrations (5 and 10 mM) decreasing the expression of these proteins at day 5 or day 9 after differentiation (Figure 4A,B). Rosiglitazone (Rosi, 2.5 µM) was used as a positive control for adipogenesis. However, it is unknown why Rosi did not induce PPARγ expression compared to control cells (DMI alone) (Figure 4A,B). Rosi significantly induced FASN and C/EBPα expression at day 5 and day 9 (Figure 4A,B).

### 2.4. Effects of Metformin on Signaling Transduction Pathways in 3T3-L1 Cells

**Figure 5:**
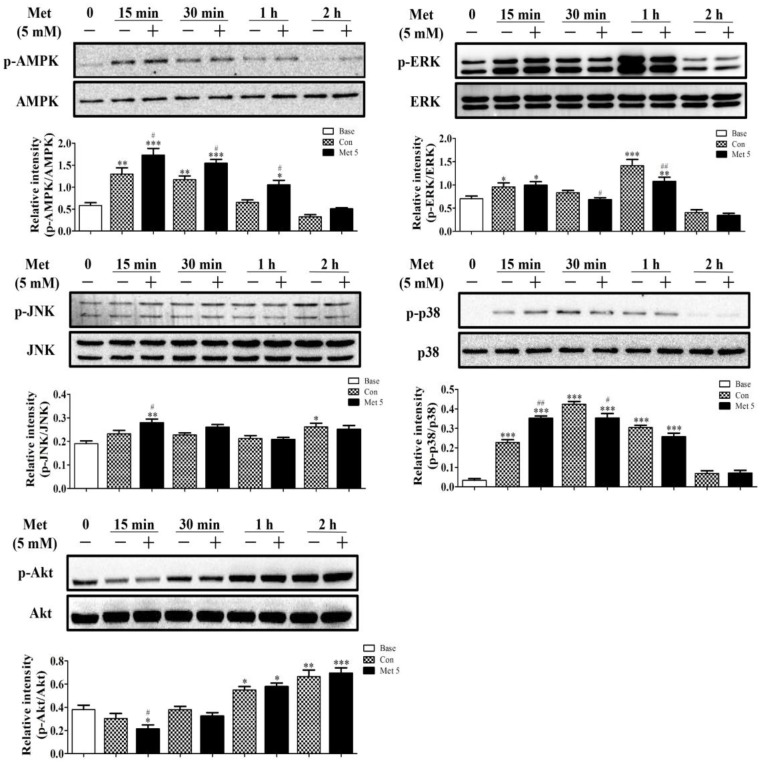
Time course of 5 mM of metformin on phosphorylation of MAPKs, Akt and AMPK in 3T3-L1 cells. Cells were cultured in DMI containing 5 mM of metformin for indicated time points; cell lysates were then collected and the expressions were determined by Western blot. Results were quantified using densitometry and normalized to total MAPKs, Akt, or AMPK accordingly (*n* = 3). “0” represents untreated cells (without DMI). Data represent the means ± SEM from three experiments. * *p* < 0.05, ** *p* < 0.01, and *** *p* < 0.001 as compared with untreated cells (time ”0”); ^#^ *p* < 0.05 and ^##^ *p* < 0.01 as compared with DMI alone treated cells at each time point (one-way ANOVA with post hoc Bonferroni’s correction).

It has been well established that both Akt kinase and the mitogen-activated protein kinase (MAPK) family, including ERK, p38, and JNK, play important roles in the process of adipogenesis. However, very few studies have examined the effects of metformin on regulating these signaling cascades during adipogenesis. Therefore, we next determined the effects of metformin on the expression of all three MAPKs and Akt phosphorylation in 3T3-L1 cells upon DMI stimulation. In addition, AMPK phosphorylation, a signaling target of metformin, was also examined. We first conducted a time course examination of the effect of 1.25 or 5 mM of metformin on regulating these proteins upon DMI stimulation at 15, 30, 60, and 120 min. As shown in Figure 5, DMI treatment with or without metformin induced AMPK phosphorylation compared to no DMI treatment as early as 15 min (*p* < 0.01). Interestingly, metformin (5 mM) treatment resulted in a significant further increase in the phosphorylation of AMPK compared to DMI alone (*p* < 0.05). The activation of AMPK by 5 mM of metformin reached its maximum at 15 min, and the activation by metformin continued until 2 h as compared to DMI only (Figure 5). Both DMI and 5 mM of metformin (with DMI) induced the phosphorylation of ERK as early as 15 min (*p* < 0.05) and reached a maximum at 1 h compared to untreated cells. However, 5 mM of metformin exhibited a significant inhibitory effect on ERK phosphorylation compared to DMI alone at 30 min (*p* < 0.05) and this inhibitory effect continued to 2 h (Figure 5). Next, DMI induced phosphorylation of p38 at as early as 15 min (*p* < 0.001) compared to no DMI treated cells; 5 mM of metformin further activated p38 but only at 15 min as compared to DMI alone (*p* < 0.01, Figure 5). However, the phosphorylation of p38 was then significantly inhibited by 5 mM of metformin compared to DMI treatment at 30 min (*p* < 0.05, Figure 5). In addition, DMI alone induced JNK activation at 2 h compared to untreated cells, while metformin significantly increased JNK activation as early as 15 min compared to DMI alone (*p* < 0.05). On the contrary, Akt phosphorylation was first inhibited by DMI with or without metformin at 15 min and then was significantly induced at 1 h (*p* < 0.05), while metformin (5 mM) significantly inhibited Akt phosphorylation at 15 min compared to DMI alone (*p* < 0.05, Figure 5A). Unfortunately, we did not find that 1.25 mM of metformin significantly deregulated the phosphorylation of the above signaling proteins from time course experiments, which suggests that pro-adipogenic effects of lower concentrations of metformin may not be mediated by these signaling cascades.

**Figure 6:**
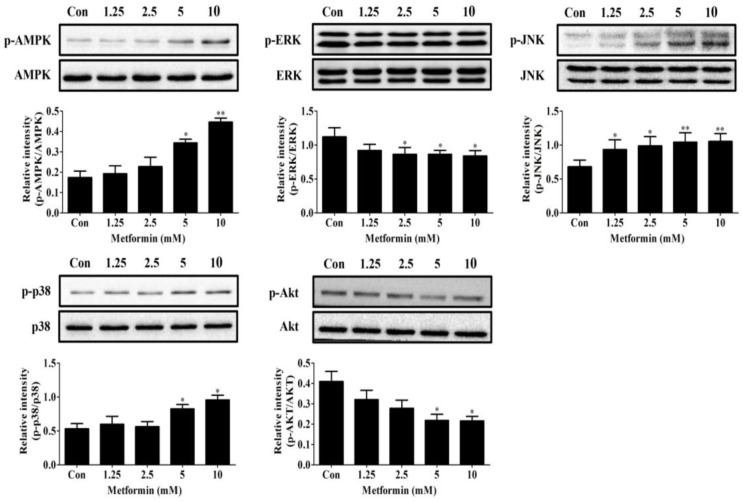
Dose response of 1.25–10 mM of metformin on phosphorylation of AMPK, Akt, and MAPKs in 3T3-L1 preadipocytes. Cells were cultured in differentiation medium containing 0, 1.25, 2.5, 5, or 10 mM of metformin for 15 min to examine AMPK, p38, JNK, and Akt activation and for 30 min to examine ERK activation. Results were quantified using densitometry analysis and normalized to total MAPKs, Akt, or AMPK accordingly (*n* = 3). Data represent the means ± SEM from three experiments. * *p* < 0.05 and ** *p* < 0.01 as compared with control (DMI alone) cells (one-way ANOVA with Dunnett’s correction).

Next, using results from the time course above, we determined the effects of different doses of metformin on the expression of these signals. As shown in Figure 6, after 15 min of treatment with metformin at various doses, the phosphorylation of AMPK and p38 were both significantly induced by higher concentrations (5 and 10 mM) of metformin (*p* < 0.05), but were not affected by treatment with lower concentrations of metformin (1.25 and 2.5 mM) as compared to DMI alone. However, metformin inhibited Akt phosphorylation in a dose-dependent manner compared to DMI alone when treated for 15 min (Figure 6). Notably, all doses of metformin induced JNK phosphorylation and reduced ERK phosphorylation (Figure 6). It is very confusing that results from the dose-course experiments did not correlate with those from the time course experiments. We speculate that the observed significant reduction of ERK and Akt phosphorylation and significant induction of JNK phosphorylation caused by lower concentrations of metformin may be due to variations among experiments. A role of JNK, Akt, and ERK in lower concentrations of metformin-induced adipogenesis could not be completely eliminated at present. Collectively, these data suggest that metformin regulates MAPKs, Akt, and AMPK signaling pathways in a very complicated manner. The exact molecular targets of the biphasic effects of metformin on adipogenic differentiation through regulating these pathways need to be further studied and identified.

### 2.5. High Concentration of Metformin Induced Inhibition of Adipogenesis Is Dependent on AMPK Activation

**Figure 7:**
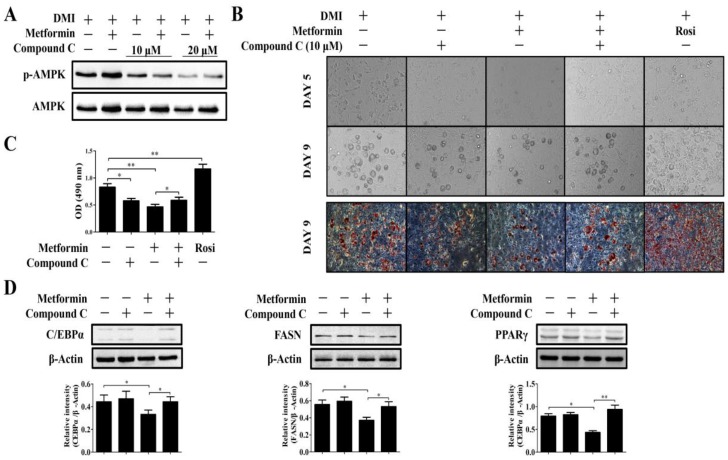
Effects of compound C on metformin-induced adipogenesis inhibition in 3T3-L1 cells. (**A**) Effects of compound C at 10 or 20 μM on AMPK phosphorylation in 3T3-L1 cells treated with or without 5 mM of metformin (*n* = 3); (**B**) Compound C at 10 μM inhibited adipogenesis, but rescued metformin-induced inhibition of adipogenesis. After pretreated with or without 10 μM compound C for 1 h, the 3T3-L1 preadipocytes were treated with DMI and metformin for the first two days, and the differentiation was continued for another 3 (Day 5) or 7 (Day 9) days, adipogenic differentiation was then determined by microscopic observation (200×) or Oil Red O staining (100×); (**C**) OD of quantified Oil Red O staining in 3T3-L1 cells (*n* = 3); (**D**) Effects of compound C on 5 mM of metformin-induced inhibition of C/EBPα, FASN, and PPARγ expression at day 9 (*n* = 3). Cells were treated in the same way as above and the protein expressions were analyzed by Western blot. Blots were quantified using densitometry analysis and results were expressed relatively after normalization to β-Actin. Data represent the means ± SEM from three experiments. * *p* < 0.05 and ** *p* < 0.01 as compared between indicated treatments in graph (one-way ANOVA with post hoc Bonferroni’s correction).

To investigate whether high concentrations of metformin-mediated AMPK activation and MAPK, p38, and JNK activation were directly required for metformin-induced adipogenesis inhibition, we treated 3T3-L1 cells with compound C, a specific AMPK inhibitor; SP600125, a specific JNK inhibitor; and SB203580, a specific p38 inhibitor. As shown in Figure 7A, 10 μM of compound C is potent enough to inhibit metformin-induced AMPK phosphorylation. Next, we examined whether inhibition of AMPK activation by compound C could reverse high concentrations of metformin-induced adipogenic inhibition in 3T3-L1 cells. To our surprise, compound C alone significantly inhibited lipid accumulation at 5 or 9 days after differentiation initiation in 3T3-L1 cells as evidenced by microscopic observation or by Oil Red O staining (Figure 7B). Consistent with the results above, metformin at 5 mM inhibited adipogenesis, while the inhibitory effect was partially prevented by compound C pretreatment (Figure 7). Both Oil Red O staining and quantification of Oil Red O by IPA confirmed that pretreatment with compound C significantly increased metformin-reduced lipid accumulation in 3T3-L1 cells (*p* < 0.05, Figure 7C). We then determined whether the expression of FASN, C/EBPα, and PPARγ correlates with the above observations. Surprisingly, we found that compound C alone had no effect on the expression of these proteins that are key regulators during adipogenesis (Figure 7D). Consistently, metformin at 5 mM inhibited the expression of FASN, C/EBPα, and PPARγ. In addition, pretreatment with compound C significantly increased the expression of metformin-induced inhibition of FASN, C/EBPα, and PPARγ proteins. However, neither SP600125 (JNK inhibitor) nor SB203580 (p38 inhibitor) increased the adipogenesis that was inhibited by 5 mM of metformin. Taken together, our data suggest that the anti-adipogenic effect of metformin at higher dosages is attributed to the activation of AMPK signaling, at least in part, in 3T3-L1 cells. However, the role of MAPKs and Akt could not be eliminated at present.

## 3. Discussion

Metformin is currently the first choice drug for the treatment of T2DM. Clinical and epidemiological studies show that obese individuals treated with metformin tend to lose more weight compared to controls [6,7]. However, the effects of metformin on adipogenic differentiation and its molecular signaling targets have not been well studied. Discrepancies on dose of action and a lack of understanding of the mechanisms underlying the effects remain, and both need to be further studied. In this study, we explored the direct effects of different concentrations of metformin on adipogenesis in 3T3-L1 preadipocytes and explored the possible molecular targets underlying the effects of metformin. Interestingly, our results suggest that metformin has a dual effect on the differentiation of 3T3-L1 cells by either promoting or suppressing adipogenesis in a concentration dependent manner, while reasons for this discrepancy are not clear at present.

Evidence from clinical studies suggest that metformin exhibits weight-lowering effects in T2DM patients, particularly when the patients are obese. However, few in vitro studies directly examined the effects of metformin on adipogenesis. After a thorough review of the literature, we found most of the studies only examined effects of one single dose of metformin on preadipocyte differentiation. Two studies examined 5 mM, and one study examined 4 mM of metformin, which significantly inhibited preadipocyte differentiation and lipid accumulation in 3T3-L1 or human preadipocytes [11,12,17]. These results are consistent with our results that metformin at 5 and 10 mM significantly inhibited 3T3-L1 preadipocyte differentiation and lipogenesis. Only a few studies examined the dose-response of metformin during adipogenesis. Tebbe et al. reported that metformin (1–5 mM) inhibited adipogenesis in a dose-dependent manner, inhibited the expression of C/EBPα, C/EBPβ, and PPARγ expression, and activated AMPK phosphorylation in 3T3-L1 cells [18]. Alexandre et al. found that metformin at 4, 8, and 16 mM reduced adipogenesis in 3T3-L1 cells in a dose-dependent manner, however, 2 mM of metformin showed no inhibitory but a slight inducing effect on adipogenesis [10]. On the contrary, Lenhard et al. reported that metformin from 100 µM to 10 mM did not show any effects on preadipocyte differentiation and lipogenesis in mouse C3H10T1/2 cells [19]. However, Chen et al. reported that metformin at 500 µM inhibited adipogenesis in C3H10T1/2 cells [20]. Unfortunately, no studies reported lower doses of metformin on adipogenesis in 3T3-L1 cells. 

To our knowledge, we are the first to examine the effects of metformin on adipogenesis at a wide range of concentrations. Our results suggest that, indeed, metformin at a nmol or µmol range had no effects on adipogenesis in 3T3-L1 cells. Surprisingly, metformin demonstrates a biphasic effect on adipogenesis at different concentrations above 1 mM: 1.25 and 2.5 mM significantly induces adipogenesis and lipid accumulation in 3T3-L1 cells, while 5 and 10 mM significantly reduces adipogenesis and lipid accumulation in cells. Despite no study reporting a direct adipogenic effect of metformin, several studies indirectly suggest a pro-adipogenic role of metformin. Expression of Sfrp5 (secreted frizzled-related protein-5) was significantly upregulated during adipogenesis [21]. Lv et al. found that, like rosiglitazone, 1 mM of metformin significantly increased the mRNA expression of Sfrp 5 gene during 3T3-L1 differentiation and adipogenesis [21]. In another study, metformin (1 mM) was found, together with insulin, to have increased human preadipocyte adipogenic capacity [22]. The concentrations of metformin used in these studies are close to the lower concentration (1.25 mM) at which metformin induced adipogenesis in our study. 

Paradoxical effects of metformin have been reported in other pathological conditions. Contradictory effects of metformin on cell angiogenesis have been reported, with some studies reporting an angiogenic activity while others showing antiangiogenic activity [23,24,25,26]. Inconsistent results also exist in the role of metformin in cancer development. A recent meta-analysis highlights much of the conflicting data between metformin use and cancer incidence [27]. In this meta-analysis, it was revealed that whereas some studies found no association between metformin use and cancer incidence or even increased risk of cancer, other studies found metformin to have a preventative role in cancer development [27]. Whether these discrepancies are due to different doses of metformin or cell model or cellular contents are unknown at present. Several clinical studies examined the efficacy and safety of dosage of metformin and found the higher the dose of metformin, the better the effects on improving glycemic control in T2DM patients [9,28]. Together with our findings, we could speculate that metformin exhibits it pharmacological effects in a dose-dependent way, and different doses may exert distinct effects. 

Adipogenesis is regulated by a complex cascade composed of up or down regulation of many transcriptional factors and a variety of signaling pathways [2,29]. *PPARγ* is a master regulator of adipocyte differentiation, which is crucial for both the initiation of differentiation and maintenance of the differentiated state during adipogenesis [16]. Members of *C*/*EBPs* transcriptional factors also participate in adipogenesis, including *C*/*EBPα*, *C*/*EBPβ*, CCAAT/enhancer binding protein γ (*C*/*EBPγ*), and *CHOP* [16]. While the pro-adipogenic *C*/*EBPβ* gene is induced early and transiently upregulated during adipocyte differentiation, *C*/*EBPα* and *PPARγ* promote terminal differentiation by transactivating downstream adipogenic genes [16,29]. In our study, we found that lower concentration of metformin (1.25 mM) significantly induced *C*/*EBPβ* gene expression during the early stage of 3T3-L1 differentiation and induced the expression of *C*/*EBPα* and *PPARγ* gene expression at a much later stage. In contrast, a higher concentration of metformin (5 mM), which inhibited adipogenesis in our results, had the opposite effect compared to 1.25 mM of metformin in 3T3-L1 cells. *CHOP* is an anti-adipogenic gene, and we found 1.25 mM of metformin significantly inhibited *CHOP* expression as early as 9 h. However, 5 mM of metformin did not significantly induce the expression of *CHOP* at any time points examined (Figure 3A). *SREBP1c* is another important transcription factor that regulates pre-adipocyte differentiation and adipogenesis [30]. In our study, we found the expression of the *SREBP1c* gene is either induced or inhibited by lower or higher concentrations of metformin, respectively, at the late stage of adipogenesis, suggesting the activation of *SREBP1c* may be more important for the maintenance of adipocyte differentiation. 

Although *PPARγ*, *C/EPBs*, and *SREBP1c* are key transcriptional regulators during adipogenesis, many other transcription factors are found to be expressed during adipogenesis and play important roles in adipocyte differentiation [16]. *KROX20*, or early growth response protein-2, has been shown to be induced during adipogenesis, and overexpression of *KROX20* promoted adipogenesis through transactivation of the *C/EBPβ* promoter in 3T3-L1 cells [31]. The Krüppel-like factors (*KLFs*) are a large family of C2H2 zinc-finger proteins which also play a role in adipocyte differentiation [16]. It has been shown that some *KLFs* are pro-adipogenic, while others are anti-adipogenic. *KLF5* is induced early during adipocyte differentiation, activating the PPARγ promoter and promoting adipogenesis [32]. In contrast, *KLF2* and *KLF7* are both anti-adipogenic factors, and *KLF2* was found to suppress the *PPARγ* promoter and thus inhibit adipogenesis [33]. Consistent with these findings, we found metformin induced the expression of *KROX20* and *KLF5* at a lower concentration (1.25 mM) but reduced the expression of these two genes at a higher concentration (5 mM). The opposite effect was observed on the anti-adipogenic gene *KLF2*. We also determined the expression of *FASN*, the key enzyme in de novo lipogenesis, and found the expression of *FASN* was significantly upregulated at both mRNA and protein levels by lower concentrations of metformin, but its expression was inhibited by higher concentrations of metformin. Interestingly, both low and high concentrations of metformin induced the expression of the thermogenesis gene *UCP-1*. Recently, Tokubuchi et al. found that metformin treatment significantly reduced fat mass, upregulated fat oxidation-related enzymes in the liver, and increased *UCP-1* expression in the brown adipose tissue of Sprague-Dawley (SD) rats as compared to untreated animals [34]. However, the exact role of metformin on thermogenesis needs to be further studied. Our data suggest that metformin could regulate a set of complex transcriptional factors and other genes that are associated with adipocyte differentiation and lipogenesis. 

Besides transcriptional regulation, adipogenesis is also mediated by multiple signaling cascades, including Wnt, transforming growth factor β (TGFβ)/bone morphogenetic protein (BMP), Notch, MAPKs, Insulin-like growth factor (IGF)/phosphatidylinositide 3-kinases (PI3K)/Akt and other signaling pathways [16]. Among these signaling cascades, the role of the MAPK family in adipogenesis has been extensively studied, but the results are quite controversial. In general, ERK may play a pro-adipogenic role during the early stage of differentiation, while the inhibition of ERK activity might be required for the maintenance of differentiation in later stages of adipogenesis [16]. Some studies suggest that p38 activation might be positively related with adipogenesis [35], but others suggest that inhibition of p38 promotes adipogenesis [36]. However, studies of the direct role of JNK in adipogenesis are scarce. In addition, the PI3K/Akt pathway is also essential for adipocyte differentiation [37]. It was reported that Akt overexpression promotes adipogenesis, and suppression of Akt has been shown to induce lipoatrophy and inhibit adipogenesis [37,38,39]. Although many studies have examined the role of MAPKs and Akt signaling cascades in adipogenesis, to our knowledge, there are very few studies about the effects of metformin on these signaling pathways during adipogenesis. In our study, we found metformin from lower to higher concentrations (1.25–10 mM) decreased Akt and ERK phosphorylation in 3T3-L1 cells. Nevertheless, only higher concentrations of metformin (5 and 10 mM) significantly increased p38 phosphorylation. Lower concentrations of metformin (1.25 and 2.5 mM) had no effect. It seems that our results are consistent with literature on adipogenesis and association with JNK, P38, and Akt signaling for the higher concentration of metformin. However, to our surprise, lower concentrations of metformin, which induced adipogenesis in our study, did not show opposite effects on the regulation of MAPKs and Akt as the higher concentrations of metformin did. Consistent with our study, Lee et al. also found that 10 mM of metformin inhibited Akt and induced JNK phosphorylation in 3T3-L1 cells [40]. Due to limited data on regulation of MAPKs and Akt by metformin from the literature, we could not ascertain whether lower concentrations of metformin (1.25 and 2.5 mM) induced adipogenesis in our results is mediated by MAPKs or Akt.

Unlike the limited studies on the association between metformin and MAPKs or Akt signaling, it has been well established that metformin exerts its pharmacological effects by activating AMPK signaling [5]. Metformin does not influence insulin secretion but rather it helps to improve the control of glycaemia by promoting glucose utilization through an AMPK-mediated stimulation of catabolism [5]. The anti-diabetic role of AMPK through increasing insulin sensitivity has been well established [41,42,43,44]. Intriguingly, it has also been reported that AMPK activation inhibits adipogenesis and metabolism of lipids [45]. In the present study, we examined whether the differentiation and lipogenesis of 3T3-L1 preadipocytes is directly regulated by metformin through the AMPK pathway. Our results indicate that metformin at higher concentrations (5 and 10 mM) increased phosphorylation of AMPK. This is consistent with the current literature. However, lower concentrations of metformin showed no effects on AMPK phosphorylation. Recently, Chen et al. found that metformin at 500 µM significantly inhibited adipogenesis in C3H10T1/2 cells, which are regulated through both AMPK-dependent and AMPK-independent way [20]. To address this apparent contradiction and to further investigate the role of AMPK in the control of adipogenesis by metformin, we examined whether an AMPK inhibitor, compound C, could counter a high concentration of metformin-induced inhibition of adipogenesis. Surprisingly, we found compound C itself significantly inhibited adipogenesis in 3T3-L1 cells based on an observational study and Oil Red O staining. However, expression of key proteins (C/EBPα, FASN, and PPARγ) were not inhibited by compound C as compared to DMI treatment alone. Interestingly, pretreatment with compound C partially prevented metformin-induced inhibition of adipogenesis. Interestingly, a literature review revealed that a number of studies also reported compound C inhibited adipogenesis. Gao et al. found that compound C significantly inhibited adipogenic differentiation of 3T3-L1 cells in a dose-dependent manner, and this inhibitory effect was primarily effective in the initial stage of differentiation [46]. Nam et al. found that compound C treatment blocked hormone-induced preadipocyte differentiation due to inhibition of mitotic clonal expansion., which is critical for 3T3-L1 pre-adipocytes to enter into the differentiation stage [47]. In a recent study, compound C was found to also significantly inhibit adipogenesis in C3H10T1/2 cells [20]. Taken together, whether metformin-induced adipogenesis inhibition is through AMPK regulation could not be elucidated at present. Our study suggests that the higher concentration of metformin-induced inhibition of adipocyte differentiation is mediated, at least in part, via AMPK activation in 3T3-L1 cells. On the contrary, lower concentrations of metformin that induced adipogenesis might not be associated with AMPK activity. These contradictory results from AMPK inhibitor studies show the need for further research. However, the physiological concentration of metformin in patients is around 20 µM as reported by Frid et al. [15] which is far less than the concentration we used in this study. Our study is, therefore, limited by a lack of clinical relevance and should to be further studied in more cellular or animal models. 

It is well established that metformin exerts its prevailing glucose-lowering effect by inhibiting hepatic gluconeogenesis, regulating lipid metabolism, and inhibiting mitochondrial complex I, which results in alteration of energy metabolism of cells [5]. However, the mechanism behind the weight-lowering effects of metformin has yet to be fully established. It was suggested that the mechanisms by which metformin contributes to weight loss may be explained through regulation of appetite, reduction in gastrointestinal absorption of carbohydrates, and reduction of leptin and ghrelin levels [7,8]. Our study is limited by only using an in vitro cell model, while the exact role and mechanisms of the biphasic effects of different concentrations of metformin on adipogenesis need to be further examined in other cell models and in vivo animal studies.

## 4. Materials and Methods 

### 4.1. Materials

Metformin, dexamethasone (DEX), rosiglitazone, 3-isobutyl-1-methylxanthie (IBMX), insulin, compound C, SP600125, and SB203580 were purchased from Sigma-Aldrich (St. Louis, MO, USA). MTT was obtained from HXBIO (Hangzhou, China). Oil Red O was purchased from Solarbio (Beijing, China). The triglyceride GPO-PAP enzymatic kit was obtained from Jiancheng Bioengineering institute (Nanjing, China). Phospho-AMPK, total AMPK, Phospho-ERK, total ERK, phosho-p38, total p38, phospho-JNK, total JNK, phospho-Akt, total Akt, FASN, PPARγ, C/EBPα, polyclonal β-Actin, and horseradish peroxidase-conjugated secondary antibodies were obtained from Cell Signaling Technology (Danvers, MA, USA). RNA extraction kit was obtained from Aidlab Biotech (Beijing, China). The iScript circular DNA (cDNA) synthesis kit and synergy brands (SYBR) master mix were purchased from Bio-Rad (Hercules, CA, USA). The bicinchoninic acid (BCA) assay kit was obtained from Pierce (Rockford, IL, USA). The Western Lightening™ Plus-ECL (Enhanced Chemiluminescence) Substrate assay kit was obtained from Perkin-Elmer (Waltham, MA, USA).

### 4.2. Cell Culture and 3T3-L1 Cell Differentiation

The 3T3-L1 mouse preadipocyte cell line was obtained from the American Type Culture Collection (ATCC, Manassas, VA, USA). Cells were seeded in DMEM supplemented with 10% fetal bovine serum (FBS) in a humidified atmosphere with 5% CO_2_ at 37 °C. At 2 days post-confluence, cells were induced to differentiation with a standard DMI containing 1 μM DEX, 0.5 mM IBMX, and 1 μg/mL insulin for 48 h. The culture medium was then changed to differentiation medium II (DMII) containing 1 μg/mL insulin every other day. To examine the effect on differentiation, metformin (dissolved in phosphate-buffered saline, PBS) at various concentrations (100 and 500 nM; 1, 10, 100, and 500 µM; and 1.25, 2.5, 5, and 10 mM) was added to the medium for the first 2 days with DMI. Rosiglitazone (2.5 μM) was used as a positive control for 3T3-L1 adipogenic differentiation. Experiments were conducted within the period of differentiation spanning from day 0 (right before DMI, D0) to day 9 (D9).

### 4.3. Cell Viability Assay

To determine the effects of metformin on cell viability, MTT assay was used. Cells were seeded in 96-well plates at a density of 1 × 10^4^ cells per well and incubated in DMEM supplemented with 10% FBS until cells reached 50% confluence. The 3T3-L1 cells were then treated with metformin at various concentrations (100 and 500 nM; 1, 10, 100, and 500 µM; and 1.25, 2.5, 5, 10, 20, and 30 mM) for 24, 48, and 72 h. MTT solution at 5 mg/mL was then added to each well and the plate was re-incubated at 37 °C for 4 h. After incubation, medium was removed and the formazan crystals were dissolved in 150 μL dimethyl sulfoxide (DMSO) for 10 min with gentle shaking. Absorbance was measured at 490 nm, and cell viability was calculated and compared with untreated cells.

### 4.4. Oil Red O Staining

After differentiation at D5 or D9, cells were washed with PBS and then fixed with 4% polyoxymethylene for 10 min. The fixed cells were stained with Oil Red O working solution (0.6% Oil Red O dye in IPA/H_2_O (3:2, *v*/*v*)) for 40 min. Subsequently, cells were washed with 60% IPA three times and photographed under a microscope. To quantify Oil Red O staining, red dye was extracted by 100% IPA and the OD was measured by a plate reader at 490 nm.

### 4.5. Triglyceride (TG) Measurement 

After differentiation, cells and medium were collected to determine intracellular and extracellular TG content using a triglyceride GPO-PAP enzymatic kit according to the manufacturer’s instruction. TG concentrations were normalized to total cellular protein concentrations as determined by the BCA method.

### 4.6. RNA Exaction and Quantitative Real-Time PCR

Total RNA was extracted from 3T3-L1 cells using an RNA extraction kit from Aidlab Biotech (Beijing, China). Both the quantity and quality of total RNA were analyzed by the Agilent Bioanalyzer 2100 system. A total of 1 µg of RNA was reverse transcribed with an iScript cDNA synthesis kit. SYBR PCR master mix was used to determine the expression of listed genes in Table 1 on the CFX96 Real-time PCR system (Bio-Rad, Hercules, CA, USA). Primers were designed with the open-sourced software Primer3Plus (Cambridge, MA, USA). The PCR conditions consisted of 40 cycles, with 5 s denaturation at 95 °C, 30 s annealing at 60 °C, and 5 s extension at 65 °C. The fold change in mRNA was calculated by the 2^−ΔΔ*C*t^ method using β-Actin as the reference gene to normalize the data for all samples. All samples were tested in duplicate, and each experiment was repeated three times. The primers used in experiments are listed in Table 1.

### 4.7. Western Blot

Total proteins were extracted from 3T3-L1 cells, and protein concentrations were quantified by the BCA method. Western blot was performed by standard method as described before [1]. The following primary antibodies were all diluted at 1:1000, including FSAN, C/EBPα, PPARγ, β-Actin, AMPK, Phospho-AMPK, Akt, Phospho-Akt, ERK1/2, Phospho-ERK1/2, p38, Phospho-p38, JNK, and Phospho-JNK. The signals were detected using an enhanced chemiluminescence substrate. ImageJ 1.41 software (Bethesda, MD, USA) was used for the calculation of OD.

### 4.8. Statistical Analysis

Quantitative data were presented as mean ± SEM of at least three independent experiments. Statistical analysis was performed using one-way ANOVA with Dunnett’s correction or Bonferroni’s correction for pairwise comparison, or two-way ANOVA with post hoc Bonferroni’s correction for multiple comparisons. All analyses were performed using GraphPad Prism 5.0 software. A value of *p* < 0.05 was considered to be statistically significant.

## 5. Conclusions

In conclusion, the present study assessed the effects of metformin on adipogenesis, finding that lower concentrations of metformin induce 3T3-L1 preadipocyte differentiation while higher concentrations of metformin inhibit adipogenesis. In addition, our data suggest that the pro-adipogenic effects of lower concentrations of metformin may not be mediated by AMPK signaling, and, at the same time, that anti-adipogenic effects of higher concentrations of metformin may be mediated, at least in part, via AMPK activation. However, the reasons underlying the discrepancy are not clear at present. More research is needed to define the role of metformin in adipocyte differentiation and the molecular targets of metformin. 

## Figures and Tables

**Table 1:** Primers used in qRT-PCR. C/EBP = CCAAT/enhancer binding protein; KLF = Krüppel-like factor; FAT/CD6 = fatty acid translocase. SREBP1c = sterol regulatory element-binding protein 1c. CHOP = C/EBP homologous protein.

Primers	Forward	Reverse
β-Actin	CTGGAACGGTGAAGGTGACA	AAGGAACTTCCTTGAACAATGCA
PPARγ	TGTCGGTTTCAGAAGTGCCTTG	TTCAGCTGGTCGATATCACTGGAG
FASN	GGAGGTGGTGATAGCCGGTAT	TGGGTAATCCATAGAGCCCAG
C/EBPα	CAAGAACAGCAACGAGTACCG	GTCACTCGTCAACTCCAGCAC
C/EBPβ	CAAGTTCCGCAGGGTGCT	CCAAGAAGACGGTGGACAA
aP2	GATGCCTTTGTGGGAACCTG	TCCTGTCGTCTGCGGTGATT
KROX20	AGAAGGTTGTGATAGGAGGTTCTC	GTTCGGATGTGAGTAGTAAGGTGG
KLF2	GCCTGTGGGTTCGCTATAAA	AAGGAATGGTCAGCCACATC
KLF5	ACCTCCGTCCTATGCCGCTAC	TCCGGGTTACTCCTTCTGTTGT
CHOP	GTCCTGTCCTCAGATGAAATTGG	GCAGGGTCAAGAGTAGTGAAGGTT
SREBP1c	CGGCTGTTGTCTACCATAAGCTG	CATAGATCTCTGCCAGTGTTGCC
UCP-1	ACTGCCACACCTCCAGTCATT	CTTTGCCTCACTCAGGATTGG
SCD-1	GGCTAGCTATCTCTGCGCTC	GAACTGCGCTTGGAAACCTG
FAT/CD36	TGGCCTTACTTGGGATTGG	CCAGTGTATATGTAGGCTCATCCA

## Abbreviations

Met	Metformin
Rosi	Rosiglitazone
DMI	Differentiation medium I
DMII	Differentiation medium II
PPARγ	Peroxisome proliferator-activated receptor
FASN	fatty acid synthase
C/EBPs	CCAAT/enhancer binding proteins
C/EBPα	CCAAT/enhancer binding protein α
C/EBPβ	CCAAT/enhancer binding protein β
FAT	fatty acid translocase
CHOP	C/EBP homologous protein
SREBP1c	sterol regulatory element-binding protein 1c
KLF2	Krüppel-like Factor 2
KLF5	Krüppel-like Factor 5
KLF7	Krüppel-like Factor 7
TGFβ	transforming growth factor β
BMP	bone morphogenetic protein
IGF	Insulin-like growth factor
PI3K	phosphatidylinositide 3-kinases
UCP-1	uncoupling protein 1
BMI	Body Mass Index
AMPK	AMP-activated protein kinase
JNK	C-Jun N-terminal kinase
ERK	Extracellular regulated protein kinases
